# The Iowa State Dental Society

**Published:** 1867-06

**Authors:** H. S. Chase


### "THE IOWA STATE DENTAL SOCIETY"

Will hold its next session at Lyons, on Tuesday, July 9th, at 7½ o'clock, P. M. There will be a session of two whole days. You are earnestly invited to attend, and take an active part in the proceedings. If you have microscopical specimens of teeth; Dental curiosities; new or improved instruments; casts of malformations, &c., &c., please bring them. Those having microscopes of 300 diameters and over, are requested to bring them. *Write out* and report any interesting cases that you may have had. *Volunteer* essays are called for.

The following Essayists have been appointed:

J. F. Sanborn, The Relation of Matter; T. P. Smith, Dental Quackery; L. C. Ingersoll, The Medical Limit of the Dentist's Sphere; H. D. Bronson, Cause and Treatment of Irregularities; R. A. Cochran, Physiology of Dentification; W. O. Kulp, Principles and Practice of Plugging Teeth; J. P. Porter, The Relative Value of Continuous Gum Work; Miss Lucy Hobbs, Treatment of Sensative Dentine; A. B. Mason, Making and Tempering Instruments; J. Hardman, Anæsthesia; H. S. Chase, Cellular Physiology.

In addition to discussion on the Essays, the following subjects will also be discussed:

Alveolar Hemorrhage; Inflammation; Peri-cementitis; Treatment of Pulp Cavities; Pathology of Dental Decay; Hereditary Influences on the Teeth; How is Decay arrested without the Aid of the Dentist; Alveolar Abscess.

Several distinguished Dentists are expected from abroad. Do not let *business* keep you from this meeting. We hope it will be the best one that we have ever had, and this is saying a great deal. It is a duty that we owe to ourselves, to our patients and to our chosen Profession to elevate and improve ourselves and each other in every thing that pertains to the Science of Dentistry.

Fraternally, Yours,

H. S. Chase,

*Corresponding Secretary.*

